# Large Outbreaks of Fungal and Bacterial Bloodstream Infections in a Neonatal Unit, South Africa, 2012–2016

**DOI:** 10.3201/eid2407.171087

**Published:** 2018-07

**Authors:** Erika van Schalkwyk, Samantha Iyaloo, Serisha D. Naicker, Tsidiso G. Maphanga, Ruth S. Mpembe, Thokozile G. Zulu, Mabatho Mhlanga, Sibongile Mahlangu, Motlatji B. Maloba, Grace Ntlemo, Kgomotso Sanyane, Dini Mawela, Nelesh P. Govender

**Affiliations:** University of Pretoria, Pretoria, South Africa (E. van Schalkwyk);; National Institute for Communicable Diseases, Johannesburg, South Africa (E. van Schalkwyk, S. Iyaloo, S.D. Naicker, T.G. Maphanga, R.S. Mpembe, T.G. Zulu, M. Mhlanga, N.P. Govender);; National Health Laboratory Service, Johannesburg (S. Mahlangu, M.B. Maloba, G. Ntlemo);; Sefako Makgato Health Sciences University, Pretoria (S. Mahlangu, M.B. Maloba, G. Ntlemo, K. Sanyane, D. Mawela);; University of the Witwatersrand, Johannesburg, (N.P. Govender);; University of Cape Town, Cape Town, South Africa (N.P. Govender)

**Keywords:** disease outbreaks, fungi, candidemia, *Candida* *krusei*, bloodstream, neonatal sepsis, infant, newborn, prematurity, fungemia, bacteremia, bacteria, cross-infection, infection control, antibacterial, antifungal, blood transfusion, intravenous, fomite, nosocomial, central venous catheter, necrotizing enterocolitis, Gauteng, South Africa

## Abstract

Candidemia is a major cause of healthcare-associated infections. We describe a large outbreak of *Candida krusei* bloodstream infections among infants in Gauteng Province, South Africa, during a 4-month period; a series of candidemia and bacteremia outbreaks in the neonatal unit followed. We detected cases by using enhanced laboratory surveillance and audited hospital wards by environmental sampling and epidemiologic studies. During July–October 2014, among 589 patients, 48 unique cases of *C. krusei* candidemia occurred (8.2% incidence). Risk factors for candidemia on multivariable analyses were necrotizing enterocolitis, birthweight <1,500 g, receipt of parenteral nutrition, and receipt of blood transfusion. Despite initial interventions, outbreaks of bloodstream infection caused by *C. krusei*, rarer fungal species, and bacterial pathogens continued in the neonatal unit through July 29, 2016. Multiple factors contributed to these outbreaks; the most functional response is to fortify infection prevention and control.

On August 4, 2014, the National Institute for Communicable Diseases (NICD) received a report of 11 neonates infected with candidemia from a university-affiliated hospital in Gauteng Province, South Africa. A large outbreak of candidemia caused by *Candida krusei* ensued over 4 months in the neonatal unit. We investigated to identify the possible source and mode of transmission of the outbreak, to identify risk factors for the development of candidemia, and to recommend control measures. After this outbreak, and despite the initial interventions, a series of >4 outbreaks of bacterial and fungal bloodstream infections (BSI) lasting until July 29, 2016, occurred. We investigated the first outbreak extensively; we report the results of this and subsequent investigations.

Candidemia may result in substantial long-term illness among hospitalized premature neonates, and reported crude mortality rates are 32%–46% (*1*–*3*). In a recent point prevalence survey among hospitalized adults and children in the United States, *Candida* was the leading pathogen causing BSI (*4*). *C. krusei*, a less common cause of BSI, is intrinsically resistant to fluconazole, a first-line antifungal agent (*5*).

Known risk factors for candidemia among neonates include very low birthweight (VLBW), prematurity, central venous catheter use, necrotizing enterocolitis (NEC), total parenteral nutrition (TPN), and prior or prolonged broad-spectrum antibacterial drug use, among others (*1*,*2*,*6*–*10*). Worldwide, outbreaks of candidemia in neonatal intensive care units (NICUs) are often caused by *C. parapsilosis* and associated with suboptimal adherence to infection prevention and control practices (*5*,*11*–*13*). In South Africa, *C. parapsilosis* is the most common *Candida* species among neonates; 2% of candidemia case-patients among all age groups test positive for *C. krusei* (*14*). 

## Methods

### Outbreak Setting

Hospital A is a 1,500-bed public-sector hospital in a semi-urban area of South Africa that serves as a referral center for 9 hospitals in 3 provinces in the region. The metropolitan area had a population of ≈3.1 million in 2014 (*15*). The infant mortality rate was estimated at 19.3/1,000 live births in Gauteng in 2014 (*16*), and the antenatal HIV prevalence was 28% (*17*).

The neonatal unit at hospital A has 55 beds, comprising 14 intensive-care beds, 20 high-care beds, and a nursery area that has 15 cots and 6 beds for surgical patients. The ward is largely of open-plan design: it has areas not fully separated by floor-to-ceiling divisions. An average of 154 patients are admitted to the unit every month. The unit is often overcrowded, and infants share cots when capacity is exceeded. Fluconazole is not routinely used as prophylaxis but was used as first-line treatment for suspected or confirmed fungemia and other invasive fungal infections before this outbreak. Amphotericin B deoxycholate was the other systemic antifungal agent available for therapeutic use; penicillin G and amikacin were used as empiric therapy for suspected bacteremia. The unit protocol requires that blood culture samples be collected for every admitted neonate at birth and for all infants in whom sepsis is suspected. A confirmatory blood culture specimen is completed before appropriate treatment is initiated. All specimens are referred to an onsite hospital laboratory with a full microbiology service.

### First Outbreak

#### Case Definition

For the outbreak investigation, we defined a case-patient as any neonate admitted to the neonatal unit during July 1–October 31, 2014, whose blood sample was positive for *C. krusei*. Any specimen positive for *C. krusei* from the same patient within 30 days of the first positive specimen was considered to be part of a single case. We defined a neonate as an infant <28 days of age; however, infants remaining in the unit or whose sample tested positive for candidemia beyond the 28th day of life were also included in this investigation.

#### Baseline Data Extraction, Confirmation of the Outbreak, and Identification of Cases

We extracted data for all cases of laboratory-confirmed candidemia during January 2012–December 2013 from the National Health Laboratory Service (NHLS) Corporate Data Warehouse (CDW), which archives demographic and laboratory data from patients whose diagnostic laboratory tests are performed by any NHLS laboratory. NICD began conducting active, laboratory-based surveillance for candidemia at enhanced surveillance sites in South Africa in 2012. Hospital A became an enhanced site in January 2014, which meant that a nurse surveillance officer at the hospital collected clinical data on a standardized case report form (including age, gestational age, gender, birthweight, mode of delivery, feeding method, and HIV exposure status and outcome) and isolates were submitted to a reference laboratory at NICD. We extracted demographic, clinical, and laboratory data for cases of candidemia from January–June 2014 from the surveillance database. We used the C2-CUSUM method (*18*) to establish a baseline of expected cases, by *Candida* species, in the unit. We detected outbreak cases through ongoing surveillance.

#### Reference Laboratory Methods

We confirmed identification and susceptibility testing of bloodstream *Candida* isolates, as previously described, with modifications (*14*). This included the use of matrix-assisted laser desorption/ionization time-of-flight mass spectrometry (Bruker Daltonics, Bremen, Germany) and sequencing of the internal transcribed spacer region of the ribosomal gene to confirm species-level identification. We did not genotype specimens.

#### Epidemiologic Studies

To determine risk factors for *C. krusei* candidemia, we conducted a retrospective cohort study. All neonates admitted for >72 hours to the neonatal unit during July 1–October 31, 2014, were included. We analyzed data from an existing ward database containing clinical data on all admitted patients and their mothers.

We collected and analyzed additional data (unavailable in the ward database) for a subset of patients by using a nested matched case–control design. We retrospectively reviewed patient and laboratory records for data pertaining to antibacterial and antifungal treatment, other medication administered (with emphasis on medication from multidose vials), intravenous fluids, TPN, blood transfusions, and laboratory parameters. Data for the presence, sites, and duration of insertion of peripheral and central venous catheters were not available. We selected 41 control-patients and 41 case-patients from the same neonatal unit who were admitted during a similar time period (±1 week) and matched by gender and birthweight (±500 g).

#### Statistical Analysis

By dividing the number of new cases by the total number of admissions to the neonatal unit during the 4-month outbreak period, we calculated the incidence of *C. krusei* and other fungal and bacterial BSI. Data on patient-days were not available.

We compared clinical and demographic characteristics of case-patients and non–case-patients in the cohort by using the Pearson χ^2^ and Fisher exact tests or Student *t*-test and Wilcoxon rank-sum test, as appropriate. We evaluated exposure variables as risk factors for candidemia by univariate analysis. Variables with p values <0.2 were included in a multivariable logistic regression model. We used conditional logistic regression to determine additional risk factors for candidemia in matched case-control pairs. We conducted all statistical analyses in Stata version 13 (StataCorp LLC, College Park, TX, USA).

#### Infection Prevention and Control Interventions

Upon recognition of the outbreak, the hospital infection control department conducted a hand hygiene campaign, and infection prevention and control (IPC) was intensified. We recommended the use of amphotericin B as the empiric antifungal agent of choice, instead of fluconazole, for all neonates with suspected candidemia. We conducted 2 IPC audits (initial, December 2014, and follow-up, March 2015), to determine whether suboptimal practices had contributed to the outbreak and to encourage improvement in IPC. We describe details of the audits in the Technical Appendix. 

We conducted 4 types of surveys during 2 IPC audit periods: during the first period, administration of IPC knowledge and perception questionnaires and targeted environmental sampling with submission of samples for fungal culture; and in both periods, a cross-sectional observational audit and observation of hand hygiene behavior. We sampled high-touch surfaces (such as procedure trolleys, intravenous fluid stands, computer monitor touchscreens and keyboards, and incubator door handles), fluids (such as TPN, a container of communal hand cream shared by staff, and a tube of water-based lubricant), contents of multidose vials (such as heparin), staff member hands, and stethoscopes.

### Subsequent Outbreaks

Ongoing surveillance identified >4 subsequent outbreaks. We performed a 1-time retrospective audit of the NHLS CDW for 2014–2015 for 3 common bacterial pathogens: *Klebsiella pneumoniae*, *Escherichia coli*, and *Enterobacter cloacae*. We compared these data with candidemia surveillance data (beginning on January 1, 2014, and ending on December 31, 2016). Results are shown in the Technical Appendix Figure.

### Ethics

NICD acquired approval for retrospective data collection for surveillance purposes and outbreak investigation activities from the Human Research Ethics Committee (Medical) of the University of Witwatersrand (reference numbers M140159 and M160667). In addition, an epidemiologic study protocol was approved (M1411112). We obtained permission to conduct the investigation from hospital A. The hospital Department of Paediatrics and Child Health granted permission for secondary data use.

## Results

### First Outbreak

**Figure 1:**
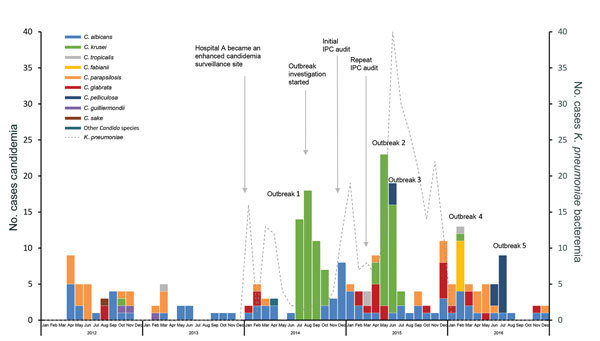
Cases of candidemia (n = 262), by *Candida* species, and bacteremia caused by *Klebsiella pneumoniae* (n = 298) in the neonatal unit at hospital A, Gauteng, South Africa, January 2012–December 2016. Individual outbreaks caused by the following *Candida* species: outbreak 1, *C. krusei*; outbreak 2, *C. krusei*; outbreak 3, *C. pelliculosa*, outbreak 4, *C. fabianii*; outbreak 5, *C. pelliculosa.* Specific points during the outbreak investigation are labeled. IPC, infection prevention and control.

In a 5-year period (January 2012–December 2016) before, during, and after the first outbreak, 262 cases of candidemia (caused by numerous *Candida* species) were detected in the neonatal unit at hospital A (Figure 1). We identified 10 different species of *Candida*; the most common was *C. krusei* (91/262; 35% of cases), followed by *C. albicans* (75/262; 29%) and *C. parapsilosis* (41/388; 16%). Cases of candidemia caused by *C. albicans* were diagnosed continually through the 5-year period; other species were identified intermittently. Before onset of the outbreak in July 2014, a single case of *C. krusei* candidemia was recorded in October 2012. During July–October 2014, of 589 neonatal admissions, 48 cases of *C. krusei* candidemia occurred, an incidence of 8.2/100 admissions. During July (n = 14), August (n = 18), and September 2014 (n = 11), *C. krusei* was the only *Candida* species detected from blood cultures in the neonatal unit. This represented a total species replacement and was above the expected baseline of 0 cases for the unit.

**Figure 2:**
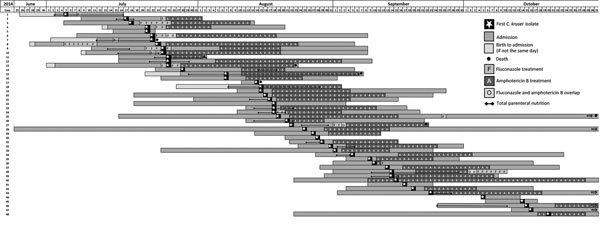
Gantt chart illustrating the timeline of an outbreak of 48 cases of *Candida krusei* bloodstream infection among neonates admitted to the neonatal unit at hospital A, Gauteng, South Africa, July 1–October 31, 2014.

The *C. krusei* index case sample was collected on July 5, 2014. Overlapping collection dates suggested a propagated outbreak with horizontal transmission of *C. krusei* among case-patients (Figure 2). The last outbreak case was confirmed from a sample collected on October 20, 2014. In samples from 48 case-patients, *C. krusei* was isolated >1 time in 29 (60%) case-patients (mean 2.5 positive isolates/case-patient). All 118 *C. krusei* isolates had amphotericin B MICs<2 µg/mL.

#### Characteristics of Outbreak Case-Patients

Among the cohort of 589 infants admitted to the neonatal unit during the 4-month outbreak period, the mean gestational age of infants with *C. krusei* candidemia (33 wk) was lower than that of infants whose samples tested negative (35 wk; p<0.001) (Table 1). Mean birthweight was also lower among positive (1,356 g) than negative (2,300 g) infants ( p<0.001). Among case-patients, 26 infants (54%) had a very low birthweight and 8 infants had an extremely low birthweight (<1,000 g). Median chronological age at onset of candidemia was 13 days (interquartile range [IQR] 7.5–17.5 days). Of 35 case-patients for whom HIV exposure status data were available, 16 (46%) had antenatal exposure to HIV; not all infants who were treated for candidemia had been tested for HIV at birth. Infants in whom candidemia was diagnosed had a longer duration of hospitalization (median 39 days, IQR 25–55 days) than did infants who tested negative (median 7 days, IQR 1–17 days; p<0.001). Of 48 infants who tested positive for candidemia, 7 died (crude case-fatality ratio 15%), compared to 62 of 538 infants who tested negative (crude case-fatality ratio 12%) (p = 0.5).

**Table 1:** Characteristics of a cohort of 589 neonates, with and without *Candida krusei* candidemia, admitted to the neonatal unit at hospital A, Gauteng, South Africa, July 2014–October 2014*

Patient characteristics	*C. krusei* candidemia, n = 48	No *C. krusei* candidemia, n = 541	Total	p value
Sex				
M	28/48 (58.3)	309/539 (57.3)	337/587 (57.4)	0.878
F	20/48 (41.7)	230/539 (42.7)	250/587 (42.6)	
Median chronological age at onset of candidemia, d (IQR)	13 (7.5–17)	NA	NA	NA
Mean gestational age at birth, wk (±SD)	33 (±3.8)	35 (±4.1)	35 (±4.1)	**<0.001**
Median birthweight, g (IQR)	1,365 (1,130–1,970)	2,300 (1,635–3,070)	2,225 (1,580–3,030)	**<0.001**
Median length of hospital stay, d (IQR)	39 (25–55)	7 (1–17)	8 (2–20)	**<0.001**
Twin infants or triplets	4/48 (8.3)	54/541 (10)	58/589 (9.8)	1.000
Born in hospital A	42/46 (91.3)	490/541 (90.6)	532/587 (90.6)	1.000
Died	7/48 (14.6)	62/538 (11.5)	69/586 (11.8)	0.468
Received antibacterial drugs during hospital stay	40/41 (97.6)	28/41 (68.3)	68/82 (82.9)	**0.001**
Median no. (IQR) antibacterial drugs received in first 13 d	3 (2–3)	2 (0–3)	2 (0–3)	**0.001**
Received TPN during hospital stay	24/40 (60)	5/41 (12.2)	29/81 (35.8)	**<0.001**
Received >1 blood transfusion during hospital stay	38/41 (92.7)	18/41 (43.9)	56/82 (68.3)	**<0.001**

*Values are no. in category/total no. (%) except as indicated. Bold indicates statistically significant values. In the No *C. krusei* candidemia group, data were unavailable for the following variables: sex (n = 2), gestational age (n = 22), birthweight (n = 1), length of hospital stay (n = 3) and death (n = 3). In the *C. krusei* candidemia group, data was unavailable for the following variables: gestational age (n = 3), birthweight (n = 2), length of hospital stay (n = 4), place of birth (n = 2). Data for the following variables were only available for a subset of patients from the nested case-control study (cases: n = 41, controls: n = 41): antibacterial drugs during hospital stay, number of antibacterial drugs in first 13 d, TPN during hospital stay and blood transfusions.  IQR, interquartile range; NA, not applicable; TPN, total parenteral nutrition.

#### Risk Factors for *C. krusei* Candidemia

After adjustment for possible confounders in the multivariable regression model, infants diagnosed with NEC were 3 times more likely to develop candidemia than those who tested negative (adjusted odds ratio [aOR] 3.1, 95% CI 1.4–6.7) (Table 2). Neonates weighing 1,000–1,500 g at birth were 6 times more likely to have candidemia than those who had a birthweight >2,500g (aOR 6.1, 95% CI 2.1–17.2). Infants who had extremely low birthweight also had a higher risk for candidemia (aOR 6.5, 95% CI 1.9–21.6). In addition, having been admitted to the unit during July and August was associated with positive test results for candidemia (Table 2).

**Table 2:** Univariate and multivariable logistic regression analysis of factors associated with candidemia caused by *Candida krusei* among infants admitted to the neonatal unit at hospital A, Gauteng, South Africa, July 1–October 31, 2014

Characteristics	Candidemia positive, no. in category/total no. (%)	Univariate analysis			Multivariable analysis	
		OR (95% CI)	p value		aOR (95% CI)	p value
Sex						
M	27/336 (58.7)	Reference			Reference	
F	19/249 (41.3)	0.9 (0.5–1.8)	0.857		0.9 (0.4–1.7)	0.671
Gestational age at birth, wks†						
<28	3/27 (6.7)	3.1 (0.7–12.1)	0.111		ND	ND
28–31	18/99 (40.0)	5.4 (2.3–12.6)	**<0.001**		ND	ND
32–36	15/209 (33.3)	1.9 (0.8–4.5)	0.141		ND	ND
≥37	9/229 (20.0)	Reference			ND	ND
Birthweight, g						
<1,000	8/44 (17.4)	8.7 (2.8–26.7)	**<0.001**		6.5 (1.9–21.6)	**0.002**
1,000–1,499	16/87 (34.8)	8.9 (3.3–23.5)	**<0.001**		6.1 (2.1–17.2)	**0.001**
1,500–1,999	11/120 (23.9)	4.0 (1.4–11.1)	**0.008**		3.4 (1.1–10.0)	**0.023**
2,000–2,499	5/93 (10.9)	2.2 (0.6–7.6)	0.193		2.5 (0.7–8.8)	0.139
≥2,500	6/242 (13.0)	Reference			Reference	
Necrotizing enterocolitis						
No	31/521 (67.4)	Reference			Reference	
Yes	15/65 (32.6)	4.8 (2.3–9.4)	**<0.001**		3.1 (1.4–6.7)	**0.005**
HIV exposed						
No	19/314 (54.3)	Reference			ND	ND
Yes	16/178 (45.7)	1.5 (0.7–3.1)	0.226		ND	ND
Month admitted						
July	21/152 (45.7)	8.9 (2.6–30.6)	**<0.001**		9.3 (2.5–33.1)	**0.001**
August	15/128 (32.6)	7.4 (2.0–26.2)	**0.002**		8.6 (2.3–31.5)	**0.001**
September	7/137 (15.2)	3.0 (0.7–11.9)	0.117		3.5 (0.8–14.4)	0.080
October	3/170 (6.5)	Reference			ND	ND
Underlying conditions						
Respiratory						
No	9/195 (19.6)	Reference			ND	ND
Yes	37/390 (80.4)	2.2 (1.0–4.6)	0.043		ND	ND
Cardiovascular						
No	25/387 (58.1)	Reference			ND	ND
Yes	18/149 (41.9)	2.0 (1.0–3.8)	0.035		ND	ND
Jaundice					ND	ND
No	17/313 (37.0)	Reference			ND	ND
Yes	29/274 (63.0)	2.1 (1.0–3.9)	0.023		ND	ND
Mother's antenatal care						
None	13/76 (28.2)	Reference			ND	ND
1–5 visits	26/392 (56.5)	0.3 (0.1–0.8)	**0.004**		ND	ND
6–10 visits	7/116 (15.2)	0.3 (0.1–0.9)	**0.018**		ND	ND
>10 visits	0/3 (0)	1			ND	ND
Mother's educational level						
<Grade 10	7/134 (16.7)	Reference			ND	ND
≥Grade 10	35/426 (83.3)	1.6 (0.7–3.8)	0.255		ND	ND
*Bold typeface indicates statistically significant values. Variables with a p value of <0.2 in the univariate analysis were added to a multivariable model and only variables that remained in the final model are displayed in the last 2 columns. Sex was included in the multivariable model as an *a* *priori *confounder. OR, odds ratio; aOR, adjusted OR; NA, not applicable; ND, no data (variables were not included in the final multivariable model). †World Health Organization classification.						

Case-patients and controls received a median of 3 (IQR 2–5) antibacterial drugs during their entire hospital stay. During the first 13 days after admission (a censored time-point corresponding to the median age of onset of candidemia), case-patients received a median of 3 (IQR 2–3) antibacterial drugs, whereas controls received a median of 2 (IQR 0–3) antibacterial drugs (p = 0.001). Of 41 case-patients, 37 (90%) received courses of antifungal therapy; 6 of these occurred before the first positive culture of *C. krusei* (fluconazole, n = 4; amphotericin B, n = 2). Of the 4 case-patients who received fluconazole, 3 were subsequently given amphotericin B, and 1 case-patient received 1 dose of fluconazole as prophylaxis on the day of surgery, 7 days before she had positive *C. krusei* culture results. 

Among 40 case-patients for whom nutritional source data were available, 24 received TPN during their hospital stay; 19 had started TPN before the first positive culture for *C. krusei* (median 4 days, IQR 3–7 days). Having received TPN at any point during hospitalization (aOR 14.1, 95% CI 1.3–143.6) and having received furosemide after blood transfusion (aOR 12.0, 95% CI 1.1–139.5) were associated with having candidemia. The number of antibacterial drugs received was not associated with candidemia in the regression model. We found no difference in median duration of TPN between cases (6 days, IQR 4–9.5 days) and controls (3 days, IQR 2–11 days) (p = 0.6).

#### Evaluation of IPC Interventions

At the time of the audit, conducted almost 2 months after the first outbreak ended, the patient census was 12% above the unit’s capacity. General cleanliness and handwashing facilities were adequate, but ventilation was poor. Isolation facilities were inadequate. A period of interrupted municipal water supply reportedly occurred in June 2014. Staff hand hygiene compliance was 76% (72 actions performed/95 opportunities). Although we isolated other bacterial and fungal species from surfaces, solutions, and staff hand samples, we were unable to find a source of *C. krusei* in the environment.

### Subsequent Outbreaks

During April–July 2015, another large outbreak consisting of 41 identified cases of *C. krusei* candidemia occurred (Figure 1, outbreak 2). Three cases of candidemia caused by *Candida pelliculosa* were retrospectively identified; these cases occurred during the second *C. krusei* outbreak in June 2015 (Figure 1, outbreak 3). Because this species had not been identified in the neonatal unit before, this cluster constituted an outbreak. Similarly, in February 2016, 7 cases of candidemia caused by *Candida* (*Cyberlindnera*) *fabianii* were detected (Figure 1, outbreak 4). In June 2016, another 8 cases of candidemia caused by *C. pelliculosa* occurred (Figure 1, outbreak 5). During January 2014–December 2015, a total of 298 cases of *K. pneumoniae* bacteremia occurred in the neonatal unit, with an overall incidence of 8/100 admissions. We retrospectively identified >3 outbreaks of *K. pneumoniae* bacteremia that appeared to closely precede or follow outbreaks of candidemia.

## Discussion

We have documented a large outbreak of *C. krusei* candidemia in a neonatal unit, reporting 48 cases occurring during 4 months. Candidemia-positive infants had a lower gestational age and birthweight than did infants negative for candidemia. NEC, birthweight <1500 g, administration of TPN, and blood transfusion were identified as risk factors. An environmental source of the outbreak was not identified, but infection was likely transmitted among infants by contact with healthcare workers and fomites.

Nosocomial outbreaks caused by other *Candida* species (mostly *C. parapsilosis*) have been reported in NICU settings in the United States, Mexico, Taiwan, and Brazil (*5*,*11*–*13*,*19*,*20*). However, none of these outbreaks was as large as the outbreak we describe. The root causes of an outbreak spanning a 4-month period are likely multifactorial; delayed recognition of the outbreak and a slow response in implementing control measures were probable contributing factors, as were broader issues such as interrupted water supply, structural problems of the building that precluded appropriate isolation of infected infants, and overcrowding in the unit. Suboptimal IPC practices, however, are usually a major contributing factor in outbreaks of this nature. In an outbreak of *C. parapsilosis* among 3 patients in a Mexico NICU, molecular testing confirmed that the hands of a healthcare worker were a source of infection (*12*). A point source from a bottle of intravenous multielectrolyte solution was identified in a 7-case outbreak of *C. krusei* fungemia in a NICU in India (*21*). Often, the sources of such outbreaks are not found.

Of neonates infected with *C. krusei* candidemia in this outbreak, >50% had very low birthweights and were born earlier than neonates who tested negative for candidemia. This finding is in agreement with other reports highlighting prematurity and low birthweight as well-recognized risk factors for candidemia (*2*,*3*,*7*). Host factors such as an immature immune system and a fragile skin barrier predispose neonates to invasive infection. Disruption of the intestinal lining, as seen in conditions like NEC, may also facilitate invasion of *Candida* into the bloodstream (*8*,*10*). We found a clear association between NEC and candidemia in this outbreak; however, we could not establish the order of onset. We used the modified Bell’s staging criteria (*22*) to diagnose and stage NEC in this unit, but the date of onset of symptoms or diagnosis was not routinely recorded. Therefore, we were unable to determine whether NEC preceded candidemia.

Administration of TPN likely represents a critical event during which *Candida* entered the bloodstream, in addition to suboptimal adherence to IPC protocols. The possibility of contaminated TPN could not be ruled out, but is unlikely in view of the propagated nature of the outbreak. Previous studies have found a longer duration of TPN to be associated with an increased risk for candidemia in older patient populations, hypothesized to be associated with prolonged exposure to glucose and lipid-rich solution, and subsequent *Candida* biofilm formation (*23*,*24*). We did not, however, find such an association, possibly because of the low number of patients who received TPN.

It is standard practice in this neonatal unit to administer a dose of furosemide after blood is administered. As with TPN, blood transfusion is not a risk factor in itself but more likely indicates exposure to an invasive procedure or a breach in IPC.

### Source of the Outbreak

Although the source of this outbreak could not be definitively established, overcrowding and suboptimal IPC practices likely contributed to transmission of *C. krusei* (online Technical Appendix). This assumption is supported by the overlap of collection dates for the first positive specimen, suggesting a propagated outbreak, as well as subsequent outbreaks of bacterial and fungal pathogens in the unit, for which similar findings were documented. *C. krusei* has been isolated from healthcare workers’ hands, hospital surfaces, and medical devices in previous studies (*25*,*26*). Although *C. krusei* was not isolated from the environment in our investigation, propagation on hands or fomites was the probable mode of transmission in this outbreak.

### Limitations

This outbreak investigation had several limitations. First, delayed recognition and initiation of a response limited the team’s ability to intervene in a timely manner. The outbreak response team had limited jurisdiction to become involved without appropriate permission from the hospital authorities; such permission to conduct an investigation was not obtained until October 2014. Second, our secondary analysis of routine ward clinical data was limited by the variables originally collected. Although we obtained additional data from patient and laboratory records, the retrospective nature of data collection meant that data were inevitably incomplete. Nonetheless, we were able to assess associations between well-established risk factors and candidemia in both epidemiologic studies. Third, because the investigation involved a closed population with a limited number of appropriately matched controls admitted to the unit during the outbreak period, the case–control study was statistically underpowered to detect true differences between the 2 groups. In addition, because identification of laboratory-confirmed cases is dependent on specimen collection practices and blood cultures have low sensitivity as a diagnostic test method, we may have misclassified potential case-patients as controls and therefore underestimated associations between risk factors and the development of candidemia. Fourth, we compiled the IPC audit after the outbreak was over, thereby reducing the probability of isolating the causative pathogen in the environment or identifying the source of the outbreak. Information on the exact location and relocation of infants within the ward was not available. We were also not able to assess staff allocations and determine which staff members were allocated to care of infants. Although we assessed the action of performing hand hygiene, we did not measure the effectiveness of those actions. We did not evaluate invasive procedures, such as administration of TPN or blood transfusions and practices around central or peripheral intravenous line maintenance.

### Recommendations Made after the Outbreak Investigation

As a result of the outbreak investigation, we re-emphasized adherence to IPC protocols at all opportunities and made further detailed recommendations (online Technical Appendix). Active surveillance for candidemia has continued at this hospital. Although there were recurrent outbreaks, response has improved.

## Conclusions

Multiple factors contributed to this outbreak of *C. krusei* candidemia and the series of subsequent outbreaks, the most critical being suboptimal adherence to IPC practices at the point of patient care. This investigation highlights the need for early detection and timely interventions in outbreaks of this nature. We did not attempt to report the resolution of a single outbreak, because contributing factors have been and are still present in this neonatal unit. Like many healthcare facilities in low- and middle-income countries, hospital infrastructure and maintenance, access to reliable water and sanitation services, and broader healthcare system and socioeconomic issues contribute to a scenario ripe for outbreaks of this magnitude to occur. A proactive approach to prevention of neonatal sepsis, with a focus on IPC and antimicrobial stewardship, is needed in this unit.

Technical Appendix Description and outcomes of the infection prevention and control audit of the neonatal unit of Hospital A, Gauteng, South Africa, during December 2014, to determine if suboptimal practices contributed to fungal and bacterial infections during the 2012–2016 outbreaks.
