# Supplementary material for: Large Outbreaks of Fungal and Bacterial Bloodstream Infections in a Neonatal Unit, South Africa, 2012–2016
Source: Emerg Infect Dis. 2018 Jul;24(7):1204–12. doi: 10.3201/eid2407.171087 (PMC6038734; doi:10.3201/eid2407.171087)
Supplement: Technical Appendix — Description and outcomes of the infection prevention and control audit of the neonatal unit of Hospital A, Gauteng, South Africa, during December 2014, to determine if suboptimal practices contributed to fungal and bacterial infections during the 2012–2016 outbreaks. [file 17-1087-Techapp-s1.pdf]

Article DOI: https://doi.org/10.3201/eid2407.171612

Large Outbreaks of Fungal and Bacterial Bloodstream Infections in a Neonatal Unit, South Africa, 2012–2016

Technical Appendix

Infection Prevention and Control Audit

Methods

We conducted an infection prevention and control audit to determine if suboptimal practices had contributed to the outbreak and to improve Infection Prevention and Control (IPC) knowledge and practices in the neonatal unit. The audit was done on 2 consecutive days in December 2014 and was comprised of 4 components: 1) cross-sectional ward audit by using an observational checklist; 2) observation of hand hygiene behavior by using a World Health Organization (WHO) tool (*1*); 3) completion of IPC knowledge and perception questionnaires by a convenient sample of healthcare workers; and 4) targeted environmental sampling, and submission of samples for fungal culture. We conducted a follow-up audit in March 2015.

The cross-sectional audit was conducted by using a checklist, which was based on an existing National Health Laboratory Service standard operating procedure for conducting IPC audits. The checklist included the following sections: patient care equipment and procedures, handwashing facilities, toilet facilities, sluice room and waste disposal, medication and infusates, feeds, common use products, staff tea room or kitchen facilities, isolation facilities, and general equipment and facilities. On 2 different days, 3 checklists were completed by independent observers. The observers completed the checklist by direct observations and questions posed to unit staff.

Observation of hand hygiene practice was performed using the WHO hand hygiene observation tool included in the My Five Moments for Hand Hygiene (*1*). Independent observers conducted 5 observation sessions lasting 20 minutes each, including 1 session during a

nightshift. Observers were assigned different times during the day of the audit and each observed 3–4 healthcare workers in the ward during performance of their routine duties. Opportunities for hand hygiene (actions which require either handwashing or using an alcohol-based hand rub) were recorded with a corresponding action (i.e., handwashing, hand rubbing or a missed opportunity).

A standardized WHO "Hand Hygiene Knowledge Questionnaire for Healthcare Workers" was administered to a convenient sample of staff on duty in the ward during the 2 audit days. A WHO hand hygiene perception survey was also administered to staff during the audit period (*1*).

Targeted environmental sampling was performed by applying sterile dry cotton swabs (without transport medium) to "high-touch surfaces" or visibly dirty areas in the unit (such as procedure trolleys, IV fluid stands, touchscreens and buttons of monitors, handles of incubator doors). We evaluated 14 areas in the unit. Hand imprints of staff, and imprints of doctors' stethoscopes and stethoscopes on the ward trolleys were taken. Samples of medication from different multi-dose vials were collected. Samples of common-use products were collected, such as total parenteral nutrition (TPN), a container of communal hand cream shared by staff and a tube of water-based lubricant. All samples were submitted to the National Institute for Communicable Diseases for fungal culture.

We compiled data from the IPC checklists using a structured questionnaire. Hand hygiene observation forms were analyzed and compliance calculations performed as recommended by the tool guidelines. Compliance (%) was calculated as the number of hand hygiene actions performed divided by the number of hand hygiene opportunities observed ×100 and stratified by professional category and indication. Completed hand hygiene knowledge and perception surveys were entered into an electronic database using Epi Info version 7 (Centers for Disease Control and Prevention, Atlanta, GA, USA) and described accordingly.

Results

At the time of the audit, conducted almost 2 months after the outbreak was over, there was noticeable commitment to improve IPC practices in the ward. General cleanliness and handwashing facilities appeared adequate. Handwashing facilities were adequate with elbow-

operated taps at sinks in each cubicle. Povidone iodine (7.5%) solution was available at every sink. Posters displaying hand hygiene techniques were visible on the walls. Examination gloves were available at the entrance to each cubicle and each incubator had a dedicated container of 70% alcohol-based hand rub.

A total of 62 infants (112% bed occupancy) were admitted in the neonatal unit on the day of the audit. We observed the unit to be hot and the ventilation system was not functional. Thermometers monitoring ambient temperature in the ward were not functional. The distance between incubators/cots ranged from 45–145 cm. Procedure trolleys and other surfaces were cleaned using a chlorine-based solution. Adequate personal protective equipment (PPE) was donned by majority of staff. A doctor was observed not wearing gloves or practicing hand hygiene between collecting blood samples from infants.

Several medications from multi-dose vials were used in the ward. A new needle and syringe were used every time the solution was drawn up for a new patient. Multi-dose vials were fitted with vial access devices.

The unit had a milk kitchen with separate “clean” and “unclean” areas. Cleanliness was good. Formula feeds were ordered from the central milk kitchen where it was prepared under sterile conditions and stored in a dedicated milk fridge in the milk kitchen. Donor milk was sterilized and stored in sterile containers in the milk fridge. Mothers expressed breast milk into plastic sterilized cups in a different ward. Expressed milk was not stored. TPN was ordered from the pharmacy and stored in the medication fridge. Sterile procedures were observed when administering the TPN and there was no sharing of feeds.

We observed 95 hand hygiene opportunities; 24 handwashing actions, and 48 hand rub opportunities. Overall, hand hygiene compliance was 76% (72 actions/95 opportunities), with professional nurses performing the best of the 4 observed staff categories (92%) and doctors performing the poorest (60%). Indication-related hand hygiene compliance was the best after touching patient surroundings (100%, n = 2), before touching a patient (82%, n = 22) and after touching a patient (82%, n = 18). The total follow-up hand hygiene compliance was 74%.

Thirteen healthcare workers were interviewed after training: 10 professional nurses (77%), 2 nursing students (15%), and 1 medical doctor (8%). IPC training had been attended by 92% (12/13) of the surveyed healthcare workers. Hand hygiene knowledge was scored at 58%.

No isolates resembling *C. krusei* were cultured from the environmental dry swabs and hand or stethoscope imprints. There was scanty bacterial and fungal growth from the multi-use vial and TPN specimen, but no growth from the water-based lubricant or hand cream samples.

Reference

1. World Health Organization. WHO Guidelines on Hand Hygiene in Health Care—tools for evaluation and feedback 2009 [updated 2009; cited December 3, 2014]. http://www.who.int/gpsc/5may/tools/evaluation_feedback/en/

Technical Appendix Table. Recommendations made after the investigation of an outbreak of candidemia caused by *Candida krusei*, followed by a series of candidemia and bacteremia outbreaks in the neonatal unit of Hospital A, Gauteng, South Africa*

Area to be addressed	Short-term recommendations	Short-to-long-term recommendations
Infection prevention and control	Adherence to local, national and international IPC protocols<br>Intensive training of staff on the importance of consistent and effective practice of IPC, in particular hand hygiene among doctors	Hospital IPC team to conduct more frequent hand hygiene observations and unannounced spot checks, with feedback to clinicians and neonatal unit staff<br>IPC incentive programs and positive reinforcement
Clinical	Implementation of IPC care bundles, such as a central line-associated BSI (CLA-BSI) care bundle if central lines are used<br>Judicious use of antimicrobial agents in the neonatal unit (both antifungal and antibacterial drugs)<br>Clinicians reminded to maintain a high index of suspicion of candidemia in premature neonates with low birthweight and concomitant NEC and those exposed to invasive procedures	Development or adoption of a formal antimicrobial stewardship program
Administrative	Diversion of mothers in pre-term labor, to reduce referrals to the neonatal unit and therefore minimizing overcrowding (this was attempted, but due to a lack of district hospitals in the area that can share the patient load, diversion was achieved for a few hours only)<br>Increasing the staff complement	Strengthening of neonatal services at surrounding hospitals
Infrastructural	Repair of ventilation system	Structural problems to be addressed to ensure more bed space<br>Construction of an adequate isolation area

*IPC, infection prevention and control; BSI, bloodstream infections.

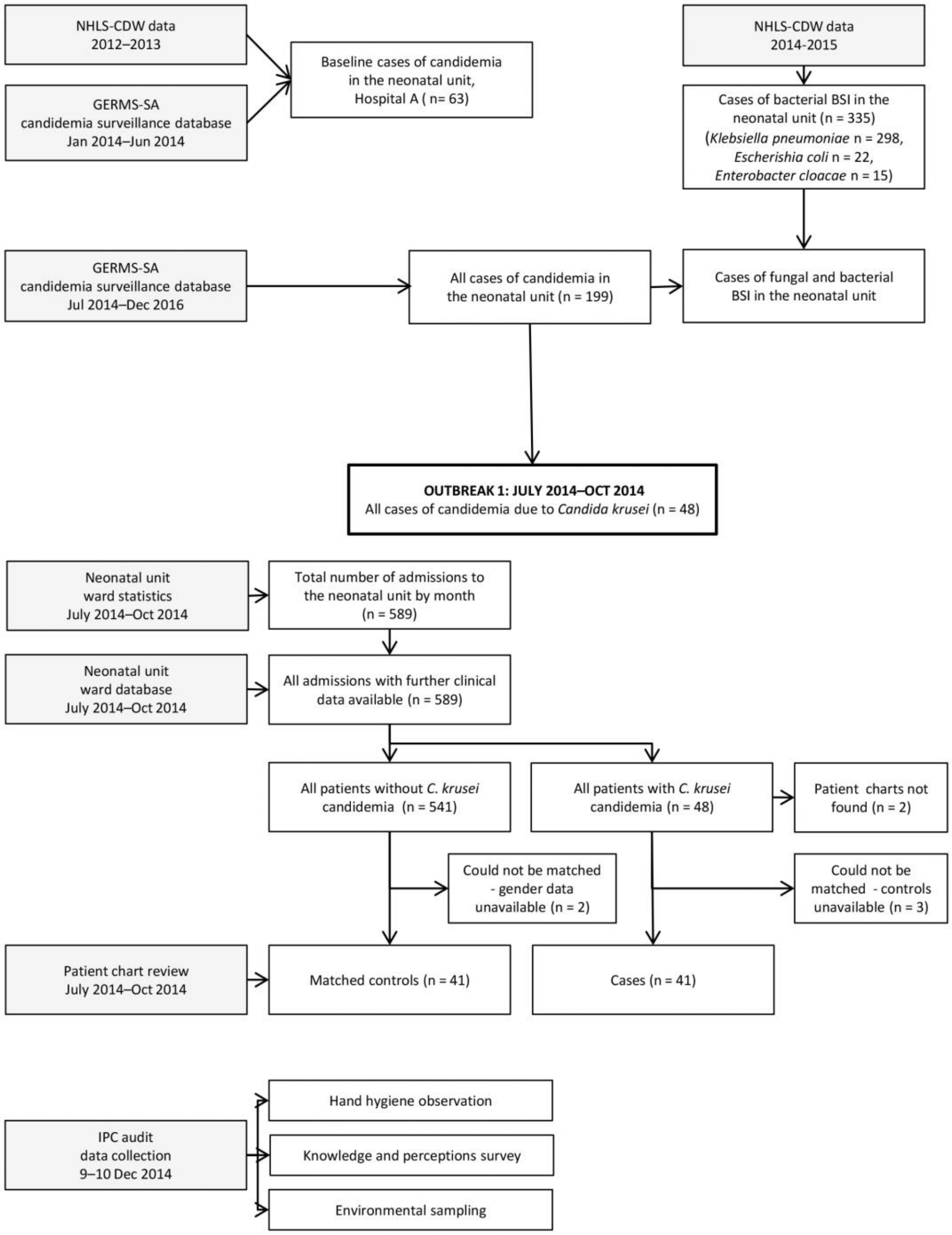

Technical Appendix Figure. Flowchart of sources and combination of data used in the investigation of an outbreak of candidemia caused by *Candida krusei* at Hospital A, Gauteng, South Africa, 2014–2016. Shaded boxes indicate data sources. NHLS-CDW, National Health Laboratory Service Corporate Data Warehouse; BSI, bloodstream infection; IPC, infection prevention and control.
